# Infantile Diarrhœa

**Published:** 1898-11-05

**Authors:** 


### INFANTILE DIARRHŒA.

The treatment of infantile diarrhœa affords an example of the support given by the conclusions of bacteriologists to the observations of clinical observers, for both are agreed as to the utility of evacuants in the treatment of this disease. The diarrhœa is so urgent a symptom, and is apparently so intimately connected with the collapse from which the patients suffer, that the inclination too often is, at all risks, to stop this distressing condition. Experienced practitioners, however, have long preached castor oil or calomel rather than astringents, and the conclusions of bacteriologists point fully in the same direction. Dr. Ashby, opening a debate on "Summer Diarrhœa," at the Clinical Society of Manchester, said that it was the universal experience that a close, hot atmosphere maintained during the whole twenty-four hours, for days together, was the indirect cause of epidemics of infantile diarrhœa, the prolonged heat supplying the conditions necessary for the increased growth and virulence of various organisms which are liable to find their way into commercial cow's milk. It is, however, by the toxins elaborated by these micro-organisms, and their absorption into the blood, that the more serious symptoms—the fever, the depression of the heart, the suppression of urine, the drowsiness, and the injurious effects upon all the tissues of the body—are produced, by which a fatal issue is so often ushered in. Locally, the toxalbumins set up an enteritis which chiefly affects the large bowel and the lower end of the ileum, but it is the absorption of the toxins that is the cause of the more serious and fatal results. In regard to treatment, then, the first thing is the evacuation of the contents of the stomach and alimentary canal, so as to clear out the microbes and all their poisonous products. The vomiting which is so often present is no doubt salutary, and may be assisted by warm water if the child will take it, or the stomach may be washed out. The latter proceeding is urgently called for in cases with no vomiting and with much collapse. Calomel is the best evacuant, and if there is no vomiting half a grain or one grain may be given and repeated once; but if there is much vomiting, so that an uncertain amount of the drug may be returned, small doses of one-sixth to one-tenth of a grain should be given and repeated every half-hour. Meanwhile, all milk must be stopped, and its place taken by barley water, white of egg in sugar water, chicken tea, or whey given in small quantities at a time. Brandy also may be given if required. So-called disinfectants to the intestinal canal have proved very disappointing, as indeed was only to be expected from what we know of the strength of disinfectants requisite to produce any definite effect upon micro-organisms outside the body. Far better is it to clear away the fermenting material, and for this purpose washing out the lower bowel is useful in addition to the use of drugs. To relieve griping and pain small doses of opium may be given, but their object is to act as sedatives and not to prevent the purging, an object for which they are too often wrongly used. If anything in the nature of a disinfectant is desired, bismuth nitrate in doses of five to 15 grains every two or three hours is as useful as any.

